# Sex comparisons in muscle sympathetic nerve activity and arterial pressure oscillations during progressive central hypovolemia

**DOI:** 10.14814/phy2.12420

**Published:** 2015-06-24

**Authors:** Robert Carter III, Carmen Hinojosa-Laborde, Victor A Convertino

**Affiliations:** US Army Institute of Surgical Research, JBSA Fort Sam HoustonHouston, Texas

**Keywords:** Baroreflex activity, blood Loss, gender, lower body negative pressure

## Abstract

Increased tolerance to central hypovolemia is generally associated with greater sympathoexcitation, high-frequency oscillatory patterns of mean arterial pressure (MAP), and tachycardia. On average, women are less tolerant to central hypovolemia than men; however, the autonomic mechanisms governing these comparisons are not fully understood. We tested the hypothesis that women with relatively high tolerance (HT) to central hypovolemia would display similar physiological reserve capacity for sympathoexcitation and oscillations in MAP at presyncope compared to HT men. About 10 men and five women were exposed to progressive lower body negative pressure (LBNP) until the presence of presyncopal symptoms. Based on our previous classification system, all subjects were classified as HT because they completed at least −60 mmHg LBNP. Muscle sympathetic serve activity (MSNA) was measured directly from the peroneal nerve via microneurography and arterial pressure (AP) was measured at the finger by photoplethysmography. LBNP time to presyncope was less (*P *< 0.01) in women (1727 ± 70 sec) than in men (2022 ± 201 sec). At presyncope, average MSNA in men (50 ± 12 bursts/min) and women (51 ± 7 bursts/min) was similar (*P *= 0.87). Coincident with similar stroke volume (SV) at presyncope, women had similar MAP and heart rates. However, women had less physiological reserve capacity for SV, AP-MSNA coherence, and oscillations in the high-frequency (HF) components of arterial pressure compared to men. Contrary to our hypothesis, lower tolerance to central hypovolemia in women was not associated with sympathoexcitation, but can be explained, in part by lower physiological reserve to elicit oscillatory patterns in AP, maintenance of AP-MSNA coherence and SV when compared to men.

## Introduction

Hemorrhagic shock following trauma is a major cause of early death of ∽40% of injured civilians and accounting for >85% of deaths of military personnel injured in combat (Eastridge et al. 2011). Lower body negative pressure (LBNP) induces central hypovolemia similar to hemorrhage (Hinojosa-Laborde et al. 2013) and thus provides a mechanism for evaluating factors which affect an individual's ability to compensate to blood loss. Tolerance to central hypovolemia is defined by the failure of compensatory mechanisms to maintain blood pressure, subsequently leading to cardiovascular decompensation and syncope.

Several investigations provide evidence that women have a lower tolerance to various conditions of reduced central blood volume compared with men (Gotshall et al. 1991; Convertino 1998a; Gotshall 2000; Carter et al. 2001; Fu et al. 2004, 2005). Women have also demonstrated greater incidence of syncopal episodes during standing after spaceflight (Fu et al. 2005) and lower predicted tolerance to passive +3 Gz acceleration (Greenleaf et al. 1985).

While it is well documented that women are generally less tolerant to central hypovolemia than men, the compensatory mechanisms governing these physiological differences are not fully understood. Lower tolerance in women is generally associated with less heart rate (HR) response to carotid baroreceptor stimulation, lower baseline cardiac vagal activity and systolic blood pressure (SAP), greater decline in cardiac output (Q) induced by LBNP, increased *β*1-adrenoreceptor responsiveness, greater vasoconstriction under equal LBNP, lower levels of total circulating norepinephrine (NE) at presyncope, and lower blood volume (Convertino 1998a). However, these conclusions were based on average sex comparisons of low tolerant women to high tolerant men without consideration for individual tolerance to central hypovolemia.

There are no previous studies in which sympathetic nervous activity was measured in a cohort of women and men who all are classified as having relatively high tolerance (HT) to progressive reductions in central blood volume. Specifically, the ‘reserve’ for autonomic responses associated with blood pressure-sympathetic nerve activity (MSNA) coherence and vasoconstrictor reserves has not been reported in HT men and women. A mechanism important for compensation to hypovolemia is activation of the sympathetic nerve system because of its influence on heart rate and peripheral vasoconstriction (Rowell 1986). The progressive increase in MSNA (Yang et al. 2012), and subsequently, elevated heart rate (Convertino et al. 2012) and peripheral vascular resistance during various levels of hypovolemia (Sundlof and Wallin 1978; Rowell 1986; Pawelczyk et al. 1988; Khan et al. 2002; Kimmerly and Shoemaker 2002; Ichinose et al. 2004; Cooke et al. 2009) is well documented. In this study, we hypothesized similar activation of the sympathetic nervous system, increases in peripheral vascular resistance (PVR), physiological reserve capacity for tachycardia, oscillations in arterial pressure, and arterial pressure/MSNA coherence in women classified as HT to central hypovolemia when compared to HT men.

## Methods

### Subjects and ethical approval

Healthy, normotensive, nonsmoking subjects volunteered to participate in this study conducted at the US Army Institute of Surgical Research (Fort Sam Houston, TX). This study was conducted under a protocol reviewed and approved by the Institutional Review Board of the Brooke Army Medical Center (Fort Sam Houston, TX) and in accordance with the approved protocol. A complete medical history and physical examination were obtained on each of the potential subjects before being approved for participation. In addition, female subjects underwent a urine pregnancy test within 24 h before experimentation and were excluded if pregnant. Subjects were instructed to maintain their normal sleep pattern and refrain from exercise, alcohol, and stimulants such as caffeine and other nonprescription drugs 24 h before testing to reduce their potential acute effects on cardiovascular responsiveness. During a familiarization session that preceded each experiment, subjects received a verbal briefing and a written description of all procedures and risks associated with the experiments and were made familiar with the laboratory, the protocol, and procedures. Each subject gave written informed consent to participate in the study.

### Study design

Lower body negative pressure was used as an experimental tool to reduce central blood volume in humans. Application of negative pressure to the lower body (below the iliac crest) results in the distribution of blood away from the upper body (head and heart) to the abdomen and lower extremities, eliciting controlled, experimentally induced hypovolemia. The LBNP protocol consisted of a 5 min control period (baseline), followed by 5 min of chamber decompression at −15, −30, −45, and −60 mmHg, then additional increments of −10 mmHg every 5 min until the onset of hemodynamic decompensation (i.e., presyncope). Hemodynamic decompensation was identified by the attending investigator by a precipitous fall in systolic pressure greater than 15 mmHg, progressive diminution of systolic pressure to less than 80 mmHg, bradycardia, and/or voluntary subject termination caused by symptoms such as gray-out (loss of color vision), tunnel vision, sweating, nausea, or dizziness. Based on our previous classification system, all subjects were classified as HT because they completed at least −60 mmHg LBNP. All experiments were conducted at room temperature (21.7–24.4°C) and ambient temperature did not change during the experiment (23.05 ± 0.19°C at baseline, 23.09 ± 0.17°C at recovery; *P *= 0.85).

All subjects were instrumented for the noninvasive, continuous measurement of HR via a standard ECG, and beat-to-beat arterial pressure via infrared finger plethysmography with a Finometer blood pressure monitor (TNO-TPD Biomedical Instrumentation, Amsterdam, the Netherlands). An appropriately sized Finometer blood pressure cuff was placed on the middle finger of the left hand, which, in turn, was laid at heart level. Each subject underwent exposure to a LBNP protocol designed to test his or her tolerance to experimentally induced hypotensive hypovolemia. Stroke volumes were estimated from the Finometer using the pulse contour method(Jansen et al. 1990). MSNA was measured directly from the peroneal nerve via microneurography.

Vagal baroreflex sensitivity (BRS) was assessed via, the sequence method (Rothlisberger et al. 2003). The WinCPRS software was used to automatically detect potential sequences between the heart rate interval (RRI) and SAP signals. A valid sequence was defined as at least three sequentially decreasing SAPs with at least a 1 mmHg change per beat and associated RRIs with at least a 4 msec change per beat (Rothlisberger et al. 2003). Baroreflex gain was then estimated via linear regression (Rothlisberger et al. 2003).

Data for all parameters were averaged across the last 3 min of each LBNP stage. If a subject reached presyncope before collection of 3 min of data at a particular LBNP level, data for that level were not used; if a subject reached presyncope after collection of at least 3 min of data, data for that LBNP level were used. To quantify oscillatory rhythms, nonequidistant beat-to-beat data were first interpolated linearly and resampled at a frequency of 5 Hz. Data were then passed through a low-pass impulse response filter with a cut-off frequency of 0.5 Hz. Three-minute data sets were fast Fourier transformed with a Hanning window to obtain power spectra. Spectral power was expressed as the integrated area within the low-frequency (LF = 0.04–0.15 Hz) and high-frequency (HF = 0.15–0.4 Hz) ranges for arterial pressure and MSNA oscillations (HRV-Task-Force, 1996); individually identified burst areas were used for MSNA oscillation determination. We calculated The unit for SAP-MSNA coherence (arbitrary units) by dividing the cross-spectral densities of the two signals of interest (either SAP or diastolic arterial pressure (DAP) with MSNA) by the product of the individual autospectra, and then averaged coherence within the LF range (deBoer et al. 1987). The unit for SAP-MSNA coherence has been verified.

### Statistical analysis

From a database of 49 human LBNP experiments (Cooke et al. 2009), data from five females (age: 32 ± 19 years; height: 161 ± 6 cm; weight: 73 ± 15 kg; means ± SD) and 10 males (age: 29 ± 10 years; height: 179 ± 8 cm; weight: 81 ± 10 kg; means ± SD) were extracted and analyzed based on the completion of the LBNP protocol to the point of decompensation with an intact nerve recording. Analysis was accomplished using commercially available software (SigmaStat; Systat Software, San Jose, CA). A one-way (LBNP level) randomized block (subjects) ANOVA for repeated measures was used for comparison of outcome variables across LBNP level. If statistical differences were found, Bonferroni-corrected comparisons with baseline measurements were performed. All data are presented as means 95% CI.

## Results

Lower body negative pressure time to presyncope was 15% lower (*P *<* *0.01) in women (1727 ± 70 sec) than in men (2022 ± 201 sec). Presyncope occurred in three women at −60, one at −70, and one at −80 mmHg compared to two men at −60, two at −70, three at −80, and three at −90.

The physiological effects produced by LBNP on hemodynamics and on the neurophysiological estimates of sympathetic activity (MSNA), total peripheral resistance (TPR), and arterial pressure oscillations (SAPLF) are displayed in Table1. Baseline levels of MSNA, MAP, SAP, DAP, Q, HR, BRS, RRIHF, SAP-MSNA coherence, SAP LF (index of arterial pressure oscillations), and TPR were not statistically distinguishable between men and women (Table1). Women (79 ± 3) had lower SV compared to men (99 ± 7).

**Table 1:** Baseline and presyncope physiological data during central hypovolemia.

	Men	Women		Men	Women	
	Baseline		*P* value	Presyncope		*P* value
HR (beats/min)	61 ± 5	67 ± 6	0.58	108 ± 8	105 ± 11	0.60
MAP (mmHg)	93 ± 6	97 ± 8	0.45	63 ± 4	64 ± 4	0.17
SAP (mmHg)	130 ± 9	128 ± 11	0.29	78 ± 4	80 ± 4	0.31
DAP (mmHg)	74 ± 5	76 ± 7	0.78	56 ± 3	58 ± 4	0.40
SV (mL)	99 ± 7	79 ± 3	<0.01	42 ± 5	37 ± 6	0.10
Q (L/min)	5.9 ± 0.4	5.3 ± 0.6	0.53	4.5 ± 1.2	4.7 ± 1.1	0.41
TPR (mmHg/L/min)	15.5 ± 5.0	13.3 ± 2.8	0.21	22.5 ± 10.1	22.5 ± 3.5	0.69
RRIHF (Vagal Index)	1301 ± 680	1032 ± 704	0.61	72 ± 59	54 ± 32	0.20
BRS	16.4 ± 3.8	18.7 ± 5.3	0.53	4.6 ± 3.8	6.4 ± 2.5	0.40
SAP-MSNA coherence (arbitrary units)	0.60 ± 0.08	0.72 ± 0.07	0.17	0.64 ± 0.11	0.53 ± 0.16	0.15
SAP LF (mmHg^2^)	6.3 ± 2.9	7.1 ± 3.7	0.23	33.8 ± 21.5	23.28 ± 8.9	0.36

All values presented as mean (± 95% CI).

**Figure 1:**
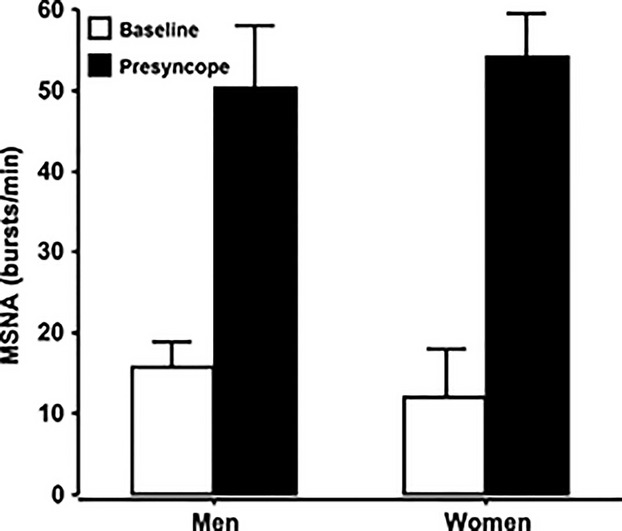
Mean (±95% CI) muscle sympathetic nerve activity at baseline (open bars) and at presyncope (closed bars) in a cohort of high tolerant men and women.

At presyncope, average MSNA values in men (50 ± 12 bursts/min) and women (51 ± 7 bursts/min) were statistically similar (*P *= 0.87) (Fig.1). Reductions in stroke volume during LBNP were associated with similar increases in MSNA and SAPLF in both men and women (Table1). At presyncope, MAP, SAP, DAP, Q, HR, BRS, RRIHF, SAP-MSNA coherence, SAP LF, and TPR were statistically indistinguishable between men and women (Table1).

The physiological changes for MAP, SAP, DAP, Q, HR, BRS, RRIHF, SAP-MSNA coherence, SAP LF, and TPR for HT men and women measured from baseline to presyncope (point of hemodynamic decompensation) are displayed in Table2. Noteworthy, men had greater physiological ‘reserves’ for SAP-MSNA coherence (0.05 ± 0.14% vs. −0.39 ± 0.28%), and SAPLF (716 ± 156 vs. 355 ± 97%; calculated as percentage increase from baseline) compared to women.

**Table 2:** Physiological ‘reserve’ during tolerance to central hypovolemia expressed as change from baseline to pre-syncope.

	Men	Women	
	Change from baseline value		*P* value
HR (beats/min)	47 ± 12	38 ± 10	0.20
MAP (mmHg)	−31 ± 9	−33 ± 8	0.45
SAP (mmHg)	−54 ± 7	−48 ± 8	0.29
DAP (mmHg)	−18 ± 5	−18 ± 7	0.78
SV (mL)	−57 ± 8	−41 ± 4	0.006
Q (L/min)	−1.6 ± 0.7	−1.4 ± 0.4	0.32
TPR (mmHg/L/min)	6.2 ± 3.9	9.2 ± 1.8	0.10
RRIHF (Vagal Index)	1229 ± 1118	1155 ± 769	0.44
BRS	16.4 ± 3.8	18.7 ± 5.3	0.53
SAP-MSNA coherence (arbitrary units)	0.05 ± 0.14	−0.39 ± 0.28	0.03
SAP LF (mmHg^2^)	27.5 ± 19	16.2 ± 6	0.09

All values presented as mean (± 95% CI).

## Discussion

Sympathetic neurovascular control plays a major role in maintaining blood pressure during central hypovolemia (Shoemaker et al. 1999; Furlan et al. 2000; Khan et al. 2002; Ichinose et al. 2006; Cooke et al. 2009; Yang et al. 2012), and subsequently tolerance to central hypovolemia. If the inherent capacity for sympathoexcitation is directly associated with tolerance to central hypovolemia, then the maximal MSNA response in HT women should be similar compared to HT men. The results of the present study supported this hypothesis in that women who met criteria for classification as individuals with HT displayed similar sympathoexcitation, increased peripheral vascular resistance, and physiological reserve capacity for tachycardia compared to their male HT counterparts. However, against expectations, similar sympathoexcitation with associated cardiovascular responses failed to translate to equal tolerance; that is, HT women demonstrated lower tolerance to reduced central blood volume when compared to the HT men.

Despite significant evidence of greater intolerance to central hypovolemia in women from this and other investigation (Convertino 1998a,b; Franke et al. 2000, 2003; Gotshall 2000; Fu et al. 2004, 2005), little is known about the role of sex differences in sympathetic reflex activation at presyncope. Our study is unique when compared with previous investigations in that the experimental design provided an opportunity to assess sympathetic responsiveness and associated blood pressure regulatory responses at presyncope in women and men who met criteria for relatively high tolerance to reduced central blood volume (Convertino et al. 2012). With this design, we are the first to report that HT women can elicit equal sympathoexcitation at the point of hemodynamic decompensation compared with HT men despite lower tolerance.

Lower levels of baseline MSNA, smaller overall increases in total MSNA, and attenuated sympathetic reflex responsiveness associated with lower tolerance to reduced central blood volume have been consistently shown in women (Hart et al. 2009, 2011; Hart and Charkoudian 2014). We assessed several measures that reflect sympathoexcitation and its associated hemodynamic responses in an attempt to explain tolerance to progressive central hypovolemia in women in our present investigation. When compared to females responses reported from previous investigations (Convertino 1998a; Franke et al. 2000; Kimmerly and Shoemaker 2002; Fu et al. 2004), women in the present study demonstrated higher tolerance and MSNA activation to central hypovolemia. Such comparisons reinforce an association between sympathoexcitation and tolerance. However, our experimental design allowed us a unique opportunity to selectively compare sympathoexcitation in women and men with relatively high tolerance. Taken together with the observation that tolerance to central hypovolemia can be increased by increasing maximal MSNA (Ryan et al. 2008; Cooke et al. 2009), the results from the present study support the notion that the onset of decompensation is a limitation to eliciting maximal sympathoexcitation rather than hypoadrenergic responses being the cause of presyncope. Furthermore, it is reasonable to suspect that differences between men and women, regardless of their individualized tolerance to hypovolemia could be associated less ability to elicit maximal sympathetic activation.

An increase in the LF component of arterial pressure (i.e., SAP) is an important hallmark of high tolerance to central hypovolemia (Rickards et al. 2011). Furthermore, increased sympathoexcitation produced by central hypovolemia enhanced the coupling between the LF components of SAP and MSNA (Cooke et al. 2009). By analyzing SAP oscillations and SAP-MSNA coherence, we addressed the hypothesis that greater oscillatory patterns might characterize increased tolerance to central hypovolemia in HT men compared with HT women. Consistent with this hypothesis, we found significantly lower ‘reserves’ for increasing SAP-MSNA coherence and SAP oscillations to central hypovolemia in women when compared to men. Thus, data from the present study support the notion that arterial pressure oscillations and their coherence with sympathoexcitation may reflect a more significant mechanism that contributes to sex differences in LBNP tolerance rather than generation of maximal sympathetic nerve activity *per se*.

Despite similarities among most of the neural and hemodynamic responses, women had smaller SV at baseline compared to men. Large reductions in SV (i.e., SV reserve) during central hypovolemia can contribute to LBNP intolerance (Levine et al. 1994; Convertino 1998a; Convertino et al. 2004). In the present study, women had smaller baseline SV and greater percentage reductions in SV from baseline to presyncope. Smaller red blood cell and total circulating blood volume could also contribute to lower SV (Convertino 1996). Therefore, in addition to blood pressure oscillations and SAP-MSNA coherence, the regulation of SV including cardiac load, size, and ‘reserve’ could contribute to the sex differences in tolerance to central hypovolemia.

Baroreceptor-mediated tachycardia provides a means to buffer transient changes in arterial blood pressure. Investigations using both human and animal models have demonstrated that carotid-cardiac baroreflex dysfunction is associated with less cardioacceleration and greater incidence of hypotension during orthostasis (Convertino and Baumgartner 1997; Convertino 1998b; Shi et al. 2003; Charkoudian et al. 2004). Although earlier onset of presyncope occurred in HT women in the present study, HR at baseline and presyncope in women did not differ from that observed in men. Previous investigations demonstrated that women with low tolerance to progressive central hypovolemia have a greater elevation in HR than HT men (Levine et al. 1994; Convertino 1998a; Convertino et al. 2004), suggesting that vagal withdrawal rather than sympathetic activation may be a more important mechanism underlying blood pressure regulation in women (Convertino 1996). The absence of sex differences in HR reserve, BRS reserve, and MSNA in the present study indicate that mechanisms of cardioacceleration did not contribute to earlier hemodynamic decompensation in women with high tolerance compared to HT men.

The results of the present study supported this hypothesis in that women who met criteria for classification as individuals with HT displayed similar sympathoexcitation, increased peripheral vascular resistance, and physiological reserve capacity for tachycardia compared to their male HT counterparts. These findings are in contrast to sex differences in general, in which a large proportion of the women will have relatively low tolerance. Nonetheless, tolerance was still lower in the women compared to the men as measured by time to syncope. Based on our analysis of the data presented in this paper, individuals use different compensatory strategies to tolerant central hypovolemia. For example, the female with the highest tolerance had one of the lower reserves for stroke volume and mSNA, but higher reserves for blood pressure (greater changes in SAP and DAP). We observed similar patterns in men. Additionally, we have similar observations in our larger data set of over 200 individuals that underwent central hypovolemia by lower body negative pressure. Unfortunately due to the limitations of maintaining mSNA until presyncope, we only have mSNA at presyncope in the subjects reported in this paper. However, based on the consistently of the responses, we are confident based on our observations and statistical analysis that these data are based on physiology, not statistical power.

Based on our previous classification system, all subjects were classified as HT because they completed at least −60 mmHg LBNP. However, the study cohort of HT women (*N* = 5) demonstrated significantly lower time to presyncope when compared to the HT men (*N* = 10).

To better understand the physiological mechanisms of women and men with equivalent tolerance to presyncope, we examined oscillations and mSNA between five women and five men with similar tolerance to central hypovolemia and the findings are the same as the larger group data (i.e., 10 males). These data suggest that individuals with physiological compensatory reserve to tolerant −60 mmHg are very unique. However, the compensatory mechanisms and physiological strategy to achieve higher levels of hypovolemia are individualized. Nevertheless, we have consistently observed that higher arterial pressure oscillations play a major role in tolerance to central hypovolemia. Both men and women reported in this study possess significantly higher arterial pressure oscillations than their lower tolerant counterparts.

In summary, the findings of the present study provided unique evidence that women who meet criteria for classification as individuals with relatively HT to central hypovolemia display similar maximal sympathoexcitation, increased peripheral vascular resistance, and physiological reserve capacity for tachycardia compared to their male HT counterparts. However, consistent with previous observations, persistently low tolerance to reduced central blood volume in females can be explained by lower physiological reserve capacity to elicit oscillatory patterns in AP, maintenance of AP-MSNA coherence and SV when compared to men.

### Limitations

It is commonly accepted that there are physiological and morphological gender differences. However, we did not measure reproductive hormone levels nor did we assess physical fitness status in our subjects. As we report, the results of independent *t*-test comparing mean MSNA between males and females were not significant. However, given that our samples were relatively small one is compelled to ask whether or not the negative result is due to low power or because the effect size is in fact small. We conducted power analyses to determine our achieved power and estimated power with increasing sample sizes, assuming the observed mean and standard deviations were constant. This analysis revealed that our study did achieve low power, in which there is only an 8% chance of detecting the observed differences with our given sample sizes. However, our additional power analyses suggest that even with a sample size of 500, 250 males and 250 females, we would only obtain power of 0.77. In fact, it would require 540 participants, 270 males and 270 females, to achieve power of 0.80, or 80% chance of detecting the observed difference. We also calculated the effect size of the difference in means between males and females using Cohen's *d*, and found an effect size of only 0.21, which is considered a small effect size. The fact that the sample size requirements to detect the observed difference are quite large and the effect size is quite small, we conclude that there is likely no significant difference in MSNA between males and females. Had we observed a moderate (0.40–0.70) or large (>0.70) effect size with low power we would have been less inclined to draw this conclusion, but this was not the case.

## Disclaimer

The opinions or assertions contained herein are the private views of the author and are not to be construed as official or as reflecting the views of the Department of the Army or the Department of Defense.

## Conflict of Interest

None declared.

## References

[b1] deBoer RW, Karemaker JM, Strackee J (1987). Hemodynamic fluctuations and baroreflex sensitivity in humans: a beat-to-beat model. Am. J. Physiol.

[b2] Carter R, Watenpaugh DE, Smith ML (2001). Genome and hormones: gender differences in physiology: selected contribution: gender differences in cardiovascular regulation during recovery from exercise. J. Appl. Physiol.

[b3] Charkoudian N, Martin EA, Dinenno FA, Eisenach JH, Dietz NM, Joyner MJ (2004). Influence of increased central venous pressure on baroreflex control of sympathetic activity in humans. Am. J. Physiol.

[b4] Convertino VA (1996). Clinical aspects of the control of plasma volume at microgravity and during return to one gravity. Med. Sci. Sports Exerc.

[b5] Convertino VA (1998a). Gender differences in autonomic function associated with blood pressure regulation. Am. J. Physiol.

[b6] Convertino VA (1998b). Gender differences in autonomic functions associated with blood pressure regualtion. Am. J. Physiol.

[b7] Convertino VA, Baumgartner N (1997). Effects of hypovolemia on aortic baroreflex control of heart rate in humans. Aviat. Space Environ. Med.

[b8] Convertino VA, Ludwig DA, Cooke WH (2004). Stroke volume and sympathetic responses to lower-body negative pressure reveal new insight into circulatory shock in humans. Auton. Neurosci.

[b9] Convertino VA, Rickards CA, Ryan KL (2012). Autonomic mechanisms associated with heart rate and vasoconstrictor reserves. Clin. Auton. Res.

[b10] Cooke WH, Rickards CA, Ryan KL, Kuusela TA, Convertino VA (2009). Muscle sympathetic nerve activity during intense lower body negative pressure to presyncope in humans. J. Physiol.

[b11] Eastridge BJ, Hardin M, Cantrell J, Oetjen-Gerdes L, Zubko T, Mallak C (2011). Died of wounds on the battlefield: casuation and implications for improving combat casualty care. J. Trauma.

[b12] Franke WD, Lee K, Graff SR, Flatau AB (2000). Effects of gender on the autonomic modulation of the cardiovascular responses to lower body negative pressure. Aviat. Space Environ. Med.

[b13] Franke WD, Johnson CP, Steinkamp JA, Wang R, Halliwill JR (2003). Cardiovascular and autonomic responses to lower body negative pressure do not explain gender differences in orthostatic tolerance. Clin. Auton. Res.

[b14] Fu Q, Arbab-Zadeh A, Perhonen MA, Zhang R, Zuckerman JH, Levine BD (2004). Hemodynamics of orthostatic intolerance: implications for gender differences. Am. J. Physiol. Heart Circ. Physiol.

[b15] Fu Q, Witkowski S, Okazaki K, Levine BD (2005). Effects of gender and hypovolemia on sympathetic neural responses to orthostatic stress. Am. J. Physiol.

[b16] Furlan R, Porta A, Costa F, Tank J, Baker L, Schiavi R (2000). Oscillatory patterns in sympathetic neural discharge and cardiovascular variables during orthostatic stimulus. Circulation.

[b17] Gotshall RW (2000). Gender differences in tolerance to lower body negative pressure. Aviat. Space Environ. Med.

[b18] Gotshall RW, Tsai P-F, Frey MAB (1991). Gender-based differences in the cardiovascular response to standing. Aviat. Space Environ. Med.

[b19] Greenleaf JE, Brock PJ, Sciaraffa D (1985). Effect of physical training in cool and hot environments on +Gz acceleration tolerance in women. Aviat. Space Environ. Med.

[b20] Hart EC, Charkoudian N (2014). Sympathetic neural regulation of blood pressure: influences of sex and aging. Physiology.

[b21] Hart EC, Charkoudian N, Wallin BG, Curry TB, Eisenach JH, Joyner MJ (2009). Sex differences in sympathetic neural-hemodynamic balance: implications for human blood pressure regulation. Hypertension.

[b22] Hart EC, Charkoudian N, Miller VM (2011). Sex, hormones and neuroeffector mechanisms. Acta Physiol.

[b23] Hinojosa-Laborde C, Shade RE, Muniz GW, Bauer C, Goei KA, Pidcoke HF (2013). Validation of lower body negative pressure as an experimental model of hemorrhage. J. Appl. Physiol.

[b24] HRV-Task-Force (1996). Task Force of the European Society of Cardiology and the North American Society of Pacing and Electrophysiology. Heart rate variability: standards of measurement, physiological interpretation, and clinical use. Circulation.

[b25] Ichinose M, Saito M, Kitano A, Hayashi K, Kondo N, Nishiyasu T (2004). Modulation of arterial baroreflex dynamic response during mild orthostatic stress in humans. J. Physiol.

[b26] Ichinose M, Saito M, Fujii N, Kondo N, Nishiyasu T (2006). Modulation of the control of muscle sympathetic nerve activity during severe orthostatic stress. J. Physiol.

[b27] Jansen JR, Wesseling KT, Settels JJ, Schreuder JJ (1990). Continuous cardiac output monitoring by pulse contour during cardiac surgery. Eur. Heart J.

[b28] Khan MH, Sinoway LI, MacLean DA (2002). Effects of graded LBNP on MSNA and interstitial norepinephrine. Am. J. Physiol. Heart Circ. Physiol.

[b29] Kimmerly DS, Shoemaker JK (2002). Hypovolemia and neurovascular control during orthostatic stress. Am. J. Physiol.

[b30] Levine BD, Giller CA, Lane LD, Buckey JC, Blomqvist CG (1994). Cerebral versus systemic hemodynamics during graded orthostatic stress in humans. Circulation.

[b31] Pawelczyk JA, Kenney WL, Kenny P (1988). Cardiovascular responses to head-up tilt after an endurance exercise program. Aviat. Space Environ. Med.

[b32] Rickards CA, Ryan KL, Cooke WH, Convertino VA (2011). Tolerance to central hypovolemia: the influence of oscillations in arterial pressure and cerebral blood velocity. J. Appl. Physiol.

[b33] Rothlisberger BW, Badra LJ, Hoag JB, Cooke WH, Kuusela TA, Tahvanainen KUO (2003). Spontaneous ‘baroreflex sequences’ occur as deterministic functions of breathing phase. Clin. Physiol. Funct. Imaging.

[b34] Rowell LB, Rowell L (1986). Adjustments to upright posture and blood loss. Human circulation: regulation during physical stress.

[b35] Ryan KL, Cook WH, Rickards CA, Lurie KG, Convertino VA (2008). Breathing through an inspiratory threshold devise improves stroke volume during central hypovolemia in humans. J. Appl. Physiol.

[b36] Shi X, Huang G, Smith SA, Zhang R, Formes KJ (2003). Aging and arterial blood pressure variability during orthostatic challenge. Gerontology.

[b37] Shoemaker JK, Hogeman CS, Sinoway LI (1999). Contributions of MSNA and stroke volume to orthostatic intolerance following bed rest. Am. J. Physiol. Regul. Integr. Comp. Physiol.

[b38] Sundlof G, Wallin BG (1978). Human muscle nerve sympathetic activity at rest. Relationship to blood pressure and age. J. Physiol.

[b39] Yang H, Cooke WH, Reed KS, Carter JR (2012). Sex differences in hemodynamic and sympathetic neural firing patterns during orthostatic challenge in humans. J. Appl. Physiol.

